# Dietary Effects of Extra Virgin Olive Oil Extracted by Ultrasound Technology or Refined Olive Oil on the Quality Traits of Pork and “Capocollo di Martina Franca” Dry-Cured Meat

**DOI:** 10.3390/ani11040954

**Published:** 2021-03-30

**Authors:** Maria Antonietta Colonna, Simona Tarricone, Francesco Giannico, Maria Selvaggi, Francesco Carriero, Pasquale Crupi, Filomena Corbo, Maria Lisa Clodoveo

**Affiliations:** 1 Department of Agricultural and Environmental Science, University of Bari Aldo Moro, 70126 Bari, Italy; mariaantonietta.colonna@uniba.it (M.A.C.); simona.tarricone@uniba.it (S.T.); 2 Department of Veterinary Medicine, University of Bari Aldo Moro, Strada Provinciale Casamassima, Km 3, 70010 Valenzano, Italy; francesco.giannico@uniba.it; 3 Salumi Martina Franca srl, 74015 Martina Franca, Italy; francesco@salumimartinafranca.it; 4 Interdisciplinary Department of Medicine, School of Medicine, University of Bari Aldo Moro, 70126 Bari, Italy; pasquale.crupi@uniba.it (P.C.); marialisa.clodoveo@uniba.it (M.L.C.); 5 Department of Pharmacy—Pharmaceutical Sciences, University of Bari Aldo Moro, 70126 Bari, Italy; filomena.corbo@uniba.it

**Keywords:** sonicated extra virgin olive oil, refined olive oil, pig, feeding, meat quality, Capocollo di Martina Franca, dry-cured meat

## Abstract

**Simple Summary:**

The “Capocollo di Martina Franca” is a traditional dry-cured pig meat product made in Apulia. Its quality depends on the raw material which, in turn, is influenced by the kind and amount of fat supplementation in the pigs’ diet. Dietary manipulation of the fatty acid composition of muscle and subcutaneous fat in pork may affect the quality and suitability of meat for processing. Vegetable oils, such as olive oil, are used for pig feeding. The extraction process of olive oil affects its content in polyphenols, which are health promoting molecules. This study was planned in order to test the effects of a diet containing extra virgin olive oil (EVOO, rich in polyphenols) extracted by ultrasound technology, in comparison with refined olive oil (ROO, devoid of polyphenols), on the quality of pig meat and capocollo. The longissimus lumborum muscle was evaluated for physical and chemical features. The capocollo was analysed in relation to its storage time after seasoning (t_1_ = 0 vs. t_2_ = +6 months). The EVOO diet improved the fatty acid profile of fresh meat and lowered the atherogenicity and thrombogenicity indices, with potential benefits for human health. Moreover, the capocollo quality was better following the EVOO diet but only immediately after ripening (t_1_ = 0).

**Abstract:**

The “Capocollo di Martina Franca” is a traditional dry-cured pig meat product made in Apulia. The dietary fat source is able to influence the lipid profile of muscle and subcutaneous fat in pork, thus affecting its nutritional and sensorial quality as well as its suitability for technological processing. The aim of the study was to evaluate the effect of a diet containing extra virgin olive oil (EVOO, 3%, as-fed basis) extracted by ultrasound technology in comparison to refined olive oil (ROO, 3%, as-fed basis) on the quality of pig meat (longissimus lumborum muscle) and capocollo in relation to its storage time after seasoning (t_1_ = 0 vs. t_2_ = +6 months). The EVOO diet lowered the concentration of myristic, palmitic, stearic and total saturated fatty acids (SFA) and increased oleic, linoleic, total monounsaturated (MUFAs), polyunsaturated (PUFAs) and n-3 and n-6 fatty acids in pig meat; moreover, the atherogenicity and thrombogenicity indices were lowered, with potential benefits for human health. The overall quality of capocollo was positively affected by the EVOO diet, although storage for 6 months after ripening levelled the protective effects of extra virgin olive oil in comparison with refined olive oil.

## 1. Introduction

The “Capocollo di Martina Franca” is a traditional dry-cured pig meat product made in the area named “Valle d’Itria” located in the Apulian region (Southern Italy). Since the XVIII century, capocollo was well known not only in Apulia but also in the Kingdom of Naples as the most representative product among the charcuterie of this territory.

Currently, the Capocollo di Martina Franca is a Slow Food Presidia project characterized by a production disciplinary aiming to protect the capocollo’s uniqueness by keeping alive the ancient butchery tradition of the area. The producers are gathered in a consortium and have strong interests to improve the quality features of the capocollo, since they aim to achieve a Protected Designation of Origin (PDO) certification that would provide guarantees to the consumer with regard to the quality and origin of the product by a system of traceability and control.

Cured pork meats have a variable and generally high fat content, ranging from 25% to 40% [1,2], that is also related to the amount and quality of lipids in fresh meat [3,4,5]. Several properties of pork cured meats depend on fat such as flavour, texture, mouthfeel, juiciness and lubricity, which affect consumers’ overall liking and acceptability [6]. However, in many countries, fat is an unpopular constituent of meat for consumers because it is considered unhealthy. Therefore, there is a growing demand to reduce fat and saturated fatty acids, both in fresh meat as well as in meat derivatives [7].

In monogastric animals, the diet is the main factor able to affect the quality of meat with regard to fat composition [8,9]. Several authors have reported that the dietary fat source influences the lipid profile of muscle and subcutaneous fat in pork, thus affecting its nutritional and sensorial quality and suitability for technological processing [8,10,11,12].

The effect of olive-derived fat in animal diets, both as oil and as fat residue in the by-products of the oil industry, such as olive pomace, has been investigated in the past [13,14,15,16]. However, little information is available on the effect that the different components that make up olive oils, i.e., the saponifiable fraction (fatty acids) and the unsaponifiable one (mainly polyphenols) may exert on the quality of fresh and processed meats. Furthermore, previous research has shown that the polyphenolic component of extra virgin olive oil may be significantly enhanced following the application of an innovative technology during its extraction process: ultrasounds. Ultrasound technology has proven useful in increasing the content of polyphenolic molecules in extra virgin olive oil [17,18,19], which are recognized by the European Food Safety Authority (EFSA) as being health-promoting components with many potential benefits [20].

This study aimed to evaluate the effect of a diet containing sonicated extra virgin olive oil rich in polyphenols in comparison to a refined olive oil on pig meat and “Capocollo di Martina Franca” dry-cured meat quality in relation to its storage time after seasoning (0 and +6 months).

## 2. Materials and Methods

### 2.1. Extra Virgin Olive Oil Extraction by Ultrasound Technology

The olive oil used for the experimental pig diets was produced in mills (1500–3000 kg/h) during 2018–2019 in industrial plants (“Le Tre Colonne” situated in Giovinazzo, Bari, Apulia, Italy). The extraction line was equipped with a two-phase centrifugal system for oil separation. The mill had a hammer crusher and the malaxers were hermetically closed. The homogeneous batches of olives of the “Coratina” cultivar were divided into samples of 800 kg each. After selection and washing, the olives were crushed. Subsequently, the crushed olive paste was directed into the Sono Heat Exchanger (SHE) and then into the malaxer. The SHE is characterized by a work capacity equal to 1500 kg/h; it is equipped with 56 transducers (100 watt and 31 kHz) able to transfer a specific energy equal to 18,000 kJ/kg. Moreover, the SHE is able to cool and heat the olive paste in relation to the environmental temperature in order to keep the temperature constant. Malaxers have been used as buffers to feed the decanter; the time of malaxation was set at 0 min for the sonicated samples and equal to 30 min for the traditional samples. The resulting extra virgin olive oil (EVOO) was collected, filtered and stored in dark glass bottles at 15 °C until use in animal feed production.

Olive pastes from the same cultivar used for EVOO extraction were submitted to the conventional industrial refinement process in order to obtain refined olive oil (ROO). The fatty acid composition (% total fatty acid methyl esters) was assessed on both oils, by applying the procedure described subsequently, and the following results were obtained, respectively for EVOO and ROO: C_16:0_, 10.58 vs. 10.60; C_16:1_, 0.50 vs. 0.52; C_18:0_, 1.67 vs. 1.72; C_18:1_, 78.88 vs. 77.92; C_18:2_, 7.44 vs. 7.07; C_18:3_, 0.76 vs. 0.77.

The content of total phenolic compounds (TPC) was analysed according to the method proposed by the International Olive Council (IOC) [21]. TPC was calculated as the sum of all the peaks in the chromatogram relative to bio-phenols [19] and was equal to 472 mg/kg for EVOO, among which hydroxytyrosol, tyrosol and their derivatives represented 93.7% of total bio-phenols (data not shown). TPC was not detectable in ROO, in accordance with Lucci et al. [20], who found that these compounds are lost following the refinement process.

### 2.2. Animals and Diets

The study was carried out in a farm located in Castellaneta (Province of Taranto, Apulia, Italy, 40°37′40″ N, 16°55′58″ E). Twenty male Large White x Landrace pigs were used in this trial. From about 80 kg live weight (±2.5 kg, average age 150 days), the animals were divided into two groups of 10 subjects each and fed a pelleted diet containing extra virgin olive oil (3% as-fed basis; EVOO) or refined olive oil (3% as-fed basis; ROO). The diets were formulated in order to be isoenergetic, isoproteic and isolipidic [22]. Pigs were housed indoors and penned into two separate collective boxes. Feed and chemical composition and the nutritive value of the feedstuffs is shown in Table 1. Pigs had ad libitum access to feed and water during the trial. Animals were cared for following the European Union guidelines (Directive 2010/63/EU).

When the pigs reached approximately a live weight of about 160 kg (±5 kg, average age 280 days), they were slaughtered in respect of the veterinary policy rules.

### 2.3. Feed Chemical Composition

Samples of each pelleted feed were ground in a hammer mill with a 1 mm screen and analysed in triplicate using the following Association of Official Analytical Chemists (AOAC) procedures [23]: dry matter (DM) (Method 934.01), ether extract (EE) (Method 920.39), ash (Method 942.05), crude protein (CP) (Method 954.01) and crude fibre (CF) (Method 945.18). Samples of each pelleted feed were used for fatty acid (FA) analysis according to the method described below for meat and capocollo FA profile.

### 2.4. Slaughtering and Meat Handling

After an on-farm fasting period of 8 h, the pigs were transported to the abattoir. They were laired for 4 h with free access to water. The pigs were electrically stunned and following exsanguination, the carcasses were scalded, dehaired and eviscerated [24].

Within 45 min after slaughter, the pH value (pH_45 min_) was measured on the longissimus lumborum (Ll) muscle using a portable instrument (Hanna Instruments HI 9025, Woonsocket, RI, USA) with a penetrating glass electrode (FC 230C, Hanna Instruments) and performing two-point calibration (pH 7.01 and 4.01). After 24 h of refrigeration at 0–4 °C, the pH was assessed again on the Ll muscle (pH_24 h_).

All the carcasses were divided at the midline into two halves; the right side was dissected in order to excise the capocollo meat cut that was immediately transported to the sausage factory for capocollo processing, while the lumbar region was taken to the laboratory to perform meat analyses. Therefore, for each group, a total of ten capocollo and ten lumbar regions were used for analysis.

### 2.5. Dry-Cured Meat Processing

The Capocollo di Martina Franca dry-cured meat was processed according to the traditional method in a sausage factory (Salumi Martina Franca srl, Martina Franca, Taranto, Apulia, Italy).

The whole piece of meat for capocollo was made of the muscles excised between the head and the beginning of the thoracic region of the pigs, corresponding to the seven cervical vertebrae, which weighed approximately 2.5–3 kg.

The capocollo was shaped, put in coarse sea salt, pepper, spices and aromatic herbs typical of the geographical area for 10–15 days, during which it was daily turned over. It was then cleaned of all the salt, washed and marinated overnight in “vincotto”, i.e., a blend of dry white wines such as Martina and Locorotondo with the addition of cooked and concentrated musts of Verdesca and Bianco d’Alessano grapes.

The capocollo was then wrapped in cotton fabric to dry for further 10–15 days. After this period, capocollo was packed into a natural gut casing and hung by a string tied on its top in order to undergo stewing, drying and the traditional seasoning process, performed by slow drying in ventilated stone rooms for about 10–15 days. Finally, it was naturally smoked by burning oak tree bark, almond peels and aromatic plants of the Mediterranean scrub and then left to mature for 6 months in dry and ventilated rooms at a temperature of 13–15 °C. At the end of the ripening period, each capocollo was split into two halves, one of which was analysed at the time (t_1_ = 0), while the other was vacuum packaged in a polyethylene bag, refrigerated at +4 °C and analysed six months later (t_2_ = +6 months); therefore, 10 samples were analysed for each group and storage time.

### 2.6. Chemical and Physical Analysis of Meat and Capocollo

The colorimetric features (L* = lightness, a* = redness and b* = yellowness) of meat and capocollo were determined using a Hunter Lab Miniscan™ XE Spectrophotometer (Model 4500/L, 45/0 LAV, 3.20 cm diameter aperture, 10° standard observer, focusing at 25 mm, illuminant D65/10; Hunter Associates Laboratory Inc., Reston, VA, USA) by taking three readings for each sample. The instrument was normalized to a standard white tile provided with the instrument before performing analysis (*Y* = 92.8, *x* = 0.3162 and *y* = 0.3322) (ASPA, 1996). The reflectance measurements were performed after the samples had oxygenated in air for at least 30 min, by which time the measurements were stable [25]. Moreover, for capocollo samples, we calculated the total colour difference (ΔE*_ab_) in order to evaluate the overall colour change between the two storage times within each group according to Larraín et al. [26].

Rheological properties of pig meat and capocollo were assessed using an Instron 5544 Universal Testing Machine (Instron Corp., Canton, MA, USA). The Warner–Bratzler shear force (WBS) of pig meat and capocollo was evaluated in triplicate using cylindrically shaped samples of one inch (25.4 mm) diameter that were sheared perpendicularly to the direction of muscle fibres (load cell 50 kg, shearing speed 200 mm/min). Peak force was expressed as kg/cm^2^ [27].

Texture profile analysis (TPA) was tested on capocollo slices of one inch (25.4 mm) diameter and 15 mm thick, using a flat steel probe of 25 mm diameter, through a double compression test elaborated by the incorporated software. The following conditions were adopted: pre-load = 0.05 N; test speed = 1 mm/s and deformation = 50%. For each experimental group, capocollo slices were evaluated in triplicate for the resistance at maximum compression during the first cycle (N), that is the force necessary to attain a given deformation, which represents the hardness of the sample at the first bite; springiness, that is the force (mm) at maximum compression during the second compression cycle, which represents the hardness of the sample at the second bite; chewiness, namely the energy required to chew a solid sample to a steady state of swallowing (N × mm) [28].

As for the assessment of pig meat cooking loss, homogeneous Ll muscle samples (about 5 cm thick) were weighed before and after cooking in a ventilated electric oven at 165 °C, until an internal temperature of 75 °C was reached in the core of the sample [9].

AOAC procedures [23] were used to analyse moisture, crude fat, protein and ash contents of Ll meat and capocollo samples.

Total lipids were extracted from the homogenized samples of meat and capocollo (100 g) using the chloroform/methanol method described by Folch et al. [29]. Fatty acids were methylated using KOH and methanol (solution 2N) [30], and the fatty acid profile was assessed using a gas chromatograph (Shimadzu GC-17A, Kyoto, Japan) with a silicate glass capillary column (70% Cyanopropyl Polysilphenylenesiloxane BPX 70 of SGE Analytical Science, length = 50 m, internal diameter = 0.22 mm and film thickness = 0.25 m). The temperature programme was as follows: 135 °C for 7 min, followed by increases of 4 °C per minute up to 210 °C and held for 2 min. The injector and detector temperatures were set at 245 and 280 °C, respectively. Helium gas was used as a carrier with a flow of 1.2 mL/min. Fatty acids were identified using a mixture of standard fatty acids (Restek Corporation, Bellefonte, PA, USA) and were expressed as percentage (wt./wt.) of total methylated fatty acids.

The food risk factors of meat and capocollo were determined by calculating the atherogenic (AI) and thrombogenic (TI) indices [31]:

AI = [(C12:0 + 4 × C14:0 + C16:0)] ÷ [ΣMUFA + Σn-6 + Σn-3];

TI = [(C14:0 + C16:0 + C18:0)] ÷ [0.5 × ΣMUFA + 0.5×Σn-6 + 3×Σn-3 + Σn-3/Σn-6];

where MUFAs are monounsaturated fatty acids.

Fatty acids were expressed as the percentage (wt./wt.) of total methylated fatty acids.

### 2.7. Lipid Oxidation

Lipid oxidation was evaluated in capocollo samples at the end of the seasoning period (S = 0) and after storage for six months (S = +6 months) in vacuum-packed bags by measuring the concentration of 2-thiobarbituric acid reactive substances (TBARS) and expressed as mg malondialdehyde (MDA)/kg meat [32].

### 2.8. Statistical Analyses

For pig meat, data were analysed for variance (ANOVA) using the General Linear Model (GLM) procedure of SAS statistical software application [33]. Data were processed by one-way ANOVA with dietary treatment as the main effect. Results are reported as least squares means and pooled SEM values. Means were compared by the Student’s *t*-test.

A mixed model for repeated measures was used to detect a possible relation between the pig diet (D; EVOO vs. ROO) and the storage period of capocollo after seasoning (S; 0 vs. 6 months) on its quality traits. The values were considered significant at *p* ≤ 0.05 and presented as least squares means and pooled SEM values. Means were compared by the Student’s *t*-test.

## 3. Results

### 3.1. Pig Meat Quality

Table 2 shows the physical and chemical features of pig meat from the longissimus lumborum muscle. The kind of olive oil in the pig diet did not affect the pH values of meat, the colour indices and the shear force (WBS).

Dietary inclusion of extra virgin olive oil determined a greater (*p* < 0.05) cooking loss as compared to that for the refined olive oil group.

The chemical composition and fatty acid profile of pig meat is shown in Table 3. The EVOO diet determined a significant (*p* < 0.05) increase of the fat content. Dietary refined olive oil significantly increased the concentration of myristic (*p* < 0.05), palmitic (*p* < 0.01) and stearic (*p* < 0.05) acids; therefore, the total SFA content was greater (*p* < 0.05).

The EVOO diet significantly (*p* < 0.05) increased the concentration of oleic and linoleic acids, total MUFAs and PUFAs and n-3 and n-6 fatty acids in meat. Furthermore, this diet markedly lowered (*p* < 0.05) the n-6/n-3 ratio and enhanced (*p* < 0.05) the PUFA/SFA ratio.

Dietary extra virgin olive oil improved the dietetic indices of pig meat since it significantly (*p* < 0.05) lowered the atherogenicity and thrombogenicity indices.

### 3.2. Capocollo di Martina Franca Dry-Cured Meat Quality

The physical features of capocollo are shown in Table 4. The ROO diet negatively affected meat colour features, especially during storage. In particular, the L value of capocollo stored for 6 months was significantly (*p* < 0.01) lower as compared to that of the EVOO group as well as to the ROO group at time 0. The a index recorded for capocollo of the ROO group after 6 months of storage was significantly higher (*p* < 0.01), thus indicating a darker redness of the capocollo, in comparison with the EVOO group as well as with the ROO at storage time 0. On the other hand, the b index was affected only by the storage time within the ROO dietary feeding treatment, which was significantly (*p* < 0.01) worsened.

The kind of olive oil used in the pig diet did not influence the WBS value of capocollo, either at time 0 or after storage. The force needed to shear the capocollo slice was markedly lower following storage (*p* < 0.05) for both dietary groups, thus indicating an effect of the storage time on the dry-cured meat texture.

As for the results related to TPA, the capocollo’s resistance was significantly (*p* < 0.01) lowered over time for both groups, while the springiness increased. The highest value of chewiness was recorded for the capocollo of the ROO group after 6 months of storage, and it was significantly (*p* < 0.01) higher as compared to the same group at time 0 as well as to the EVOO group.

Table 5 shows the results obtained from the chemical analysis of capocollo. As expected, storage for six months lowered (*p* < 0.05) the moisture content of capocollo regardless of the pigs’ diet. The MDA concentration was greater in the ROO group as compared to the EVOO diet at time 0. Storage determined an increase (*p* < 0.05) of the MDA concentration in both dietary groups.

The fatty acid profile of capocollo is shown in Table 6. Dietary refined olive oil determined a significant increase of the concentration of C18:3 c6,9,12 (-linolenic; *p* < 0.01) and C21:0 (heneicosylic acid; *p* < 0.05) at both storage times, while it lowered (*p* < 0.05) the concentration of C18:1 c9 (n-9) (oleic) and C20:2 c11, c14 (n-6) (eicosadienoic) acids. The thrombogenicity index was significantly (*p* < 0.05) lower at both storage times following the EVOO diet.

## 4. Discussion

The effect of vegetable oil supplementation in the pigs’ diet may affect the quality and nutritive value of pork meat as well as its content of bioactive compounds [8,11,34]. Indeed, in this study, the kind of olive oil used for pig feeding influenced the quality of fresh meat and capocollo dry-cured meat.

As for meat from the longissimus lumborum muscle, the pH values recorded at slaughtering and after 24 h of refrigeration were consistent with those reported by other studies [9,14,35,36]. The colour features of meat were not affected by the type of olive oil used in the pig diet, as previously found by Nuernberg et al., who tested the effects of feeding 5% linseed or olive oil in castrated and female pigs [14]. In this study, dietary extra virgin olive oil determined a greater cooking loss as compared to the refined olive oil group. Scheeder et al. reported that feeding 7% pork fat, 4.95% olive oil or 3.17% soybean oil to growing-finishing pigs did not affect pH, colour, texture or cooking loss in pork [37]. The eating quality of pork is a combination of many factors among which are tenderness and juiciness, which are also affected by the temperature and duration of cooking. Many different experimental methods have been used to cook pork, making it difficult to compare the results since the final internal temperature is not always clearly defined [28].

In this study, the chemical composition and fatty acid profile of meat were affected by the pigs’ diet. In comparison with extra virgin olive oil, refined olive oil worsened the overall fatty acid profile of fresh meat since it increased the concentration of myristic, palmitic and stearic acids. Myristic and palmitic acids are well known to have an atherogenic effect, while stearic acid is neutral in relation to the atherogenicity, although it has a documented thrombogenic effect [31].

The concentration of oleic and linoleic acids in pig meat was higher following the EVOO diet as compared to the ROO one, according to the findings reported by Ostrowska et al. [15]. Dietary extra virgin olive oil as compared to refined olive oil enhanced the concentration of total MUFAs and PUFAs in fresh meat, with benefits for human health. Furthermore, the diet containing extra virgin olive oil significantly increased the concentration of total n-3 fatty acids while it decreased the concentration of those of the n-6 series, thus improving the n-6/n-3 ratio, which was also markedly lowered. Even the PUFA/SFA ratio was greater following the EVOO diet; the values obtained in this study for both feeding treatments are below the limit of 0.45 that is recommended for human health [38].

In comparison with extra virgin olive oil, the use of refined olive oil in the pig diet negatively influenced all the capocollo meat colour features, especially during storage. Škrlep et al. reported that lower colour intensity and vividness of pork sausages was associated with higher levels of oxidation that leads to the formation of yellow-coloured polymers and myoglobin oxidation products [39].

The calculation of the ΔE*ab value has provided the following results: ΔE*ab_EVOO_ = 1.06, while ΔE*ab_ROO_ = 4.91. According to the criteria suggested by Larraín et al. [26], changes in instrumental colour measurements are visually noticeable when ΔE*ab values range between 2 and 6, although no exact threshold has been established for meat colour difference detection. Based on this statement, in our trial, only the ΔE*ab_ROO_ could be potentially visible to the average person, but further investigations would be advisable to confirm this trend. Higher stability of redness and yellowness indices in the EVOO groups may be due to the well-documented higher concentration of antioxidant molecules contained in extra virgin olive oil, including phenolic compounds [18]. Furthermore, the concentration of antioxidants in olive oil depends on the olive cultivar and extraction process, being higher when olive oil is extracted by a physical process [40]. The ultrasound technology applied during the extraction process has been proven to enhance the concentration of bioactive compounds in extra virgin olive oil [18]. However, these antioxidant compounds are lost when olive oil is extracted by solvents as occurs during the refinement process [20]. It can, therefore, be hypothesized that the lack of these compounds in ROO may have affected over time the stability of capocollo towards oxidation.

The kind of olive oil used in the pigs’ diet did not influence the WBS value of capocollo, either at time 0 or after storage for 6 months, and this value was affected only by storage. Changes of tenderness of the meat and meat products during storage have been reported in several studies as a result of the enzymatic degradation of muscle tissue [41]. This degradation is caused by proteolytic enzymes such as calpains and liposomal proteases. Moreover, the temperature of storage can affect the enzymatic degradation, as well as other factors including the following: pH, amount and degree of cross-linking of connective tissue and animal species [41]. Previous studies reported that changes in firmness and toughness in salami may be due to changes in water content that normally occur during storage [42] or to the rearing system, namely the physical exercise of animals during the fattening phase [43]. In terms of the texture profile analysis, the storage period increased the capocollo’s springiness regardless of the pigs’ diet. Capocollo from the ROO diet showed a worsened chewiness after storage in comparison with the EVOO group, thus negatively affecting capocollo eating quality. García-Esteban et al. found an increase of the hardness and chewiness of vacuum-packed dry-cured ham during storage attributable to both water content and the state of proteins [44]. Indeed, in this study, the moisture content of capocollo from the ROO group stored for six months after ripening was lower as compared to that of the EVOO group, along with a slightly higher amount of proteins, which may have affected the overall texture of capocollo during storage.

The MDA concentration was lower in the EVOO groups as compared to the ROO ones; this result confirms the protective effect of extra virgin olive oil rich in polyphenols on the prevention of lipid oxidation in capocollo. Storage contributes to a series of secondary reactions, which provoke the degradation of lipids and the development of oxidative rancidity [45]. This process is one of the major factors responsible for the gradual reduction of the sensory and nutritional quality of meats and meat products. In order to control lipid oxidation during storage, some technologies have been developed, among which is vacuum packaging by using plastic films with low permeability to oxygen. Although air is removed prior to sealing and vacuum application ensures tight contact of the low-permeability films to the meat or meat product, an accumulation of small amounts of fluid exudate occurs, as found also in our study. The residual O_2_ remaining in the package is able to determine a slow oxidation over time [45]. The MDA values recorded in this study, anyway, are quite low and far below the concentration of 2 mg MDA/kg meat, which is considered to be the limit above which rancidity is perceived by consumers [46].

## 5. Conclusions

The dietary inclusion of extra virgin olive oil extracted by ultrasound technology in the pigs’ diet improved fresh meat quality traits as well as the fatty acid composition of capocollo as compared to refined olive oil. However, storage for 6 months after ripening levelled the protective effects of extra virgin olive oil in comparison with refined olive oil on the overall shelf-life of capocollo. Further studies are required in order to evaluate whether the association of natural antioxidants to dietary extra virgin olive oil extracted by ultrasound technology is able to increase the concentration of health-promoting bioactive compounds into muscle tissues in order to prolong their presence during the storage process in charcuterie products.

## Figures and Tables

**Table 1:** Feed ingredients (g/kg, as-fed basis) and chemical composition (% dry matter) of the pelleted feeds.

Ingredients	EVOO	ROO
Corn meal	366.5	366.5
Barley	230.0	230.0
Soybean meal (43.4% CP, crude protein)	159.0	159.0
Wheat flour shorts	115.0	115.0
Wheat middlings	40.0	40.0
Sugar cane molasses	30.0	30.0
Extra virgin olive oil ^1^	30.0	-
Refined olive oil	-	30.0
Monocalcium phosphate	8.0	8.0
Limestone	14.0	14.0
Sodium chloride	4.0	4.0
Lysine	1.7	1.7
Premix ^1^	1.0	1.0
Choline	0.8	0.8
Chemical composition (% dry matter)	-	-
Moisture	10.5	10.5
Crude protein	16.5	16.5
Ether extract	6.0	6.0
Ash	5.9	5.9
Crude fibre	4.5	4.5
Metabolizable Energy (ME; MJ/kg)	12.80	12.80

^1^ One kilogram of the Premix contains: vitamin A 6000 IU, vitamin D3 800 IU, vitamin E 20 mg, niacin 6 mg, Ca D-pantothenate 4 mg, riboflavin 1.5 mg, vitamin K 1 mg, vitamin B6 1 mg, vitamin B1 0.8 mg, folic acid 0.25 mg, biotin 0.05 mg, vitamin B12 0.01 mg, choline chloride 600 mg, MnO 25.8 mg, ZnO 24.8 mg, FeSO_4_·H_2_O 76 mg, FeCO_3_·H_2_O 20.7 mg, CuSO_4_·5H_2_O 19.65 mg, Ca(IO_3_)_2_ 0.62 mg, Na_2_SeO_3_ 0.18 mg and L-lysine monohydrochloride 1.326 mg. EVOO, extra virgin olive oil; ROO, refined olive oil.

**Table 2:** Mean (±SE) physical and chemical characteristics of meat from the longissimus lumborum muscle of pigs fed a diet containing extra virgin olive oil (EVOO) or refined olive oil (ROO).

Item	EVOO	ROO	SEM ^1^	*p*-Value
pH_45 min_	6.15	6.14	0.261	0.054
pH_24 h_	5.59	5.56	0.060	0.054
L*	48.30	48.39	0.736	0.067
a*	7.39	8.04	0.393	0.172
b*	11.71	10.84	0.561	0.051
WBS (kg/cm^2^)	2.02	1.76	0.173	0.264
Cooking loss (%)	26.64	24.62	1.259	0.041

^1^ Standard error of means. L* = lightness, a* = redness and b* = yellowness. WBS, Warner–Bratzler shear force.

**Table 3:** Mean (±SE) chemical (%) and fatty acid (FA) composition (% of total FA methyl esters) of meat from the longissimus lumborum muscle of pigs fed a diet containing extra virgin (EVOO) or refined olive oil (ROO).

Item	EVOO	ROO	SEM ^1^	*p*-Value
Moisture	74.02	73.77	0.262	0.276
Protein	21.84	22.21	0.455	0.069
Fat	2.02	1.88	0.326	0.048
Ash	1.34	1.37	0.123	0.350
N-free extracts	0.78	0.87	0.069	0.789
C14:0 (myristic)	1.01	1.80	0.456	0.045
C16:0 (palmitic)	21.80	27.50	3.291	<0.01
C18:0 (stearic)	12.10	16.80	2.714	0.023
C20:0 (arachidic)	2.30	2.90	0.346	0.213
C16:1 c9 (palmitoleic)	2.30	2.04	0.058	0.112
C18:1 c9 (oleic)	43.30	35.40	4.561	0.043
C18:2 n6 (linoleic)	11.40	8.50	1.674	0.032
C18:3 n3 (α-linolenic)	0.70	0.50	0.115	0.063
C20:4 n6 (arachidonic)	3.20	2.90	0.173	1.018
C20:5 n3 (eicosapentaenoic, EPA)	0.20	0.20	0.003	2.087
C22:5 n3 (docosapentaenoic, DPA)	0.30	0.10	0.116	1.376
C22:6 n3 (docosahexaenoic, DHA)	0.07	0.05	0.012	1.769
Total SFA	37.21	49.00	6.807	0.034
Total MUFA	45.6	37.8	4.503	0.047
Total PUFA	15.87	12.25	2.090	0.032
Total n-3	1.27	0.85	0.243	0.050
Total n-6	14.60	11.40	1.848	0.042
n-6/n-3	11.50	13.41	1.103	0.027
PUFA/SFA	0.42	0.25	0.103	0.050
Atherogenic index (AI)	0.42	0.63	0.035	0.033
Thrombogenic index (TI)	1.27	1.43	0.035	0.021

^1^ Standard error of means. SFA, Saturated Fatty Acids; MUFA, Monounsaturated Fatty Acids; PUFA, Polyunsaturated Fatty Acids.

**Table 4:** Effect of the pig diet (EVOO and ROO) and of the storage period (0 and +6 months) on the physical traits of Capocollo di Martina Franca dry-cured meat.

Item	EVOO		ROO		SEM ^1^	Effects		
	0	+6 Months	0	+6 Months		D	S	D × S
L*	39.58	40.08 ^A^	40.71 ^C^	37.57 ^B,D^	1.514	0.207	0.020	<0.01
a*	9.28	8.50 ^B^	8.91 ^D^	12.01 ^A, C^	1.247	<0.01	0.013	<0.01
b*	6.78	7.30	6.27 ^D^	8.44 ^C^	0.820	0.286	<0.01	<0.01
WBS (kg/cm^2^)	3.69 ^c^	3.34 ^d^	3.88 ^c^	3.36 ^d^	0.146	0.611	0.049	0.047
Resistance (N)	8.58 ^c^	6.89 ^d^	8.21 ^c^	7.60 ^d^	1.152	0.680	0.042	0.192
Springiness (mm)	0.43 ^D^	0.83 ^C^	0.39 ^D^	0.89 ^C^	0.108	0.740	<0.01	0.199
Chewiness (N·mm)	3.69 ^D^	5.71 ^B,C^	3.20 ^D^	6.74 ^A,C^	1.757	<0.01	<0.01	<0.01

^1^ Standard error of means. L* = lightness, a* = redness and b* = yellowness. Differences between diets: ^A,B^: *p* < 0.01; differences between storage periods: ^c,d^: *p* < 0.05; ^C,D^: *p* < 0.01.

**Table 5:** Effect of the pigs’ diet (EVOO and ROO) and of the storage period (0 and +6 months) on the chemical composition and malondialdehyde (MDA) concentration of Capocollo di Martina Franca dry-cured meat.

Item	EVOO		ROO		SEM ^1^	Effects		
	0	+6 Months	0	+6 Months		D	S	D × S
Moisture	38.07 ^c^	35.90 ^d^	36.59 ^c^	34.70 ^d^	1.594	0.069	0.050	0.052
Protein	22.93	23.73	23.48	24.18	0.854	0.065	0.102	0.089
Fat	31.65	32.76	32.19	33.15	1.180	0.057	0.059	0.158
Ash	5.90	6.10	5.91	6.09	0.959	0.071	0.214	0.485
N-free extracts	1.45	1.51	1.83	1.88	0.319	0.077	0.315	0.965
MDA (mg/kg meat)	0.035 ^b,d^	0.069 ^c^	0.047 ^a,d^	0.077 ^c^	0.008	0.049	0.025	0.042

^1^ Standard error of means. Differences between diets: ^a,b^: *p* < 0.05; differences between storage periods: ^c,d^: *p* < 0.05.

**Table 6:** Effect of the pigs’ diet (EVOO and ROO) and of the storage period (0 and +6 months) of Capocollo di Martina Franca dry-cured meat on fatty acid composition.

Fatty Acid (% of Total FA Methyl Esters)	EVOO		ROO		SEM ^1^	Effects		
	0	+6 Months	0	+6 Months		D	S	D × S
C10:0 (capric)	0.061	0.056	0.074	0.074	0.008	0.210	0.445	0.394
C12:0 (lauric)	0.076	0.070	0.093	0.093	0.006	0.198	0.236	0.197
C14:0 (myristic)	1.418	1.390	1.458	1.460	0.028	0.071	0.194	0.156
C15:0 (pentadecylic)	0.037	0.032	0.052	0.052	0.005	0.062	0.133	0.152
C16:0 (palmitic)	25.613	25.446	25.734	25.989	0.591	0.124	0.834	0.321
C17:0 (margaric)	0.246	0.242	0.247	0.259	0.079	0.753	0.891	0.760
C18:0 (stearic)	15.643	15.470	16.187	16.162	0.555	0.062	0.618	0.709
C20:0 (arachidic)	0.507	0.530	0.424	0.436	0.091	0.052	0.582	0.866
C21:0 (heneicosylic acid)	0.101 ^b^	0.108 ^b^	0.198 ^a^	0.200 ^a^	0.033	0.051	0.199	0.137
C24:0 (lignoceric acid)	0.075	0.069	0.089	0.089	0.114	0.142	0.139	0.145
C16:1 c9 (n-7) (palmitoleic)	1.565	1.376	1.600	1.438	0.243	0.059	0.578	0.870
C17:1 c10 (n-9) (eptadecenoic)	0.150	0.131	0.208	0.208	0.062	0.098	0.217	0.229
C18:1 c9 (n-9) (oleic)	39.764 ^a^	40.192 ^a^	38.302 ^b^	38.404 ^b^	1.249	0.042	0.554	0.714
C18:2 c9, c12 (n-6) (linoleic)	10.068	10.121	9.929	9.920	1.195	0.686	0.961	0.938
C20:2 c11, c14 (n-6) (eicosadienoic)	0.207 ^a^	0.253 ^a^	0.069 ^b^	0.070 ^b^	0.049	0.025	0.183	0.207
C18:3 c9,12,15 (n-3) (α-linolenic)	0.692	0.710	0.635	0.636	0.095	0.060	0.771	0.799
C18:3 c6,9,12 (n-6) (γ-linolenic)	0.283 ^B^	0.164 ^B^	0.643 ^A^	0.642 ^A^	0.126	<0.01	0.185	0.194
Total n-3	0.692	0.710	0.635	0.636	0.095	0.060	0.771	0.799
Total n-6	10.558	10.538	10.641	10.632	1.126	0.893	0.994	0.965
n-6/n-3	15.257	14.842	16.757	16.717	3.825	0.144	0.988	0.452
Total SFA	43.777	43.413	44.556	44.814	0.540	0.210	0.693	0.090
Total MUFA	41.479	41.699	40.110	40.050	1.453	0.055	0.616	0.578
Total PUFA	11.250	11.248	11.276	11.268	1.136	0.787	0.982	0.938
Other acids	3.494	3.640	4.058	3.868	0.128	0.056	0.499	0.565
PUFA/SFA	0.257	0.259	0.253	0.251	0.026	0.276	0.932	0.739
Atherogenic index (AI)	0.595	0.594	0.616	0.622	0.012	0.058	0.566	0.111
Thrombogenic index (TI)	1.515 ^b^	1.494 ^b^	1.587 ^a^	1.597 ^a^	0.043	0.040	0.429	0.130

^1^ Standard error of means. SFA, Saturated Fatty Acids; MUFA, Monounsaturated Fatty Acids; PUFA, Polyunsaturated Fatty Acids. Differences between diets: ^a,b^: *p* < 0.05; ^A,B^: *p* < 0.01.

## Data Availability

The datasets used and/or analysed during the current study are available from the first author and from the corresponding author on reasonable request.
